# Conservation physiology today and tomorrow

**DOI:** 10.1093/conphys/cot033

**Published:** 2014-01-07

**Authors:** Steven J. Cooke

**Affiliations:** Editor-in-Chief, Conservation Physiology Canada Research Chair, Carleton University, Ottawa, Ontario, Canada

Some eight years ago Martin Wikelski and I started musing about how physiological knowledge and tools could be used to understand conservation problems. We were certainly not the first to do so, but we were able to codify the idea by coining the term ‘conservation physiology’ (Wikelski and Cooke, 2006), which has been embraced by many. When the leadership at the Society of Experimental Biology (SEB), including Tony Farrell, Craig Franklin, and Paul Hutchinson, embarked on a plan to launch a new journal, the topic of conservation physiology quickly rose to the top, given its timeliness and potential to engage both plant and animal researchers. That was nearly two years ago. Today I am thrilled to launch the second volume of the journal *Conservation Physiology*, published jointly by Oxford University Press (OUP) and the SEB. Here I briefly reflect on the first year (2013) of contributions to *Conservation Physiology* and consider what can be expected in 2014 and beyond.

The *Conservation Physiology* submission portal opened quietly in December of 2012 and, with negligible advertising, we started receiving submissions. The inaugural paper (Cooke *et al.*, 2013) was published in March, with the official launch of the journal occurring with great fanfare at the SEB Annual Meeting in Valencia, Spain in July. As of December 2013, Volume 1 had over 30 papers published, with another handful of accepted articles in production. The content is remarkably diverse, spanning taxa including plants (Funk, 2013), invertebrates (Timmins-Schiffman *et al.*, 2013), fish (Brix and Grosell, 2013), marine mammals (Burgess *et al.*, 2013), terrestrial mammals (Freeman *et al.*, 2013), herpetofauna (Stacy *et al.*, 2013), and birds (Milenkaya *et al.*, 2013).

Likewise, the topics are diverse, but two topical areas certainly emerge, i.e. non-invasive methods of studying disturbance and/or reproductive state in wild animals, and the effects of environmental change on organismal physiology and survival. The former topic has included contributions that review the tools available for studying the physiology of free-living whales (Hunt *et al.*, 2013) and amphibians (Narayan, 2013), as well as empirical studies that use ‘fake’ eggs with heart-rate sensors to assess human disturbance thresholds in penguins (Ellenberg *et al.*, 2013) and faecal glucocorticoid sampling to assess logging and hunting disturbance in primates (Rimbach *et al.*, 2013). The latter topic, environmental change, has included papers that examine how climate change modifies host–parasite interactions (Rohr *et al.*, 2013) and considering how thermal conditions influence the respiratory physiology of migratory Pacific salmon (Eliason *et al.*, 2013). Other environmental changes have also been considered, including modified nutrient conditions in phosphorus-limited environments and their influence on native plant diversity (Lambers *et al.*, 2013), as well as the effect of increased CO_2_ conditions on the aerobic scope of reef fish (Rummer *et al.*, 2013). A refreshing characteristic of nearly all of the content is that it extends beyond documenting ‘problems’ towards proposing and even testing possible solutions [e.g. bycatch reduction for sharks using knowledge of sensory physiology (Jordan *et al.*, 2013); refinement of seed-banking procedures for wild plants (Hay and Probert, 2013); and testing recovery protocols for fish exhausted by capture prior to release (Robinson *et al.*, 2013)]. Such solution-based approaches are urgently needed in conservation science (Salafsky *et al.*, 2002; Balmford and Cowling, 2006).

For those working in the area of conservation physiology, a number of challenges exist. The single biggest challenge is to ensure that findings are relevant to conservation practitioners (Cooke and O'Connor, 2010). In other words, although mechanistic research in conservation is important, it is equally important to ensure that it is clear what such data mean. The challenge is even greater given that physiologists tend to work at the level of the molecule, cell, or organism, while conservation practitioners and managers focus on populations and species. Reconciling those differences in organizational scale by demonstrating how physiology influences behaviour, fitness, and demography remains an important goal for conservation physiology. Nonetheless, given the ability of physiological tools to develop cause-and-effect relationships (Carey, 2005), conservation practitioners have the potential to be provided with findings that can be incorporated rapidly into conservation and management regimens. It is my hope that the journal and its content will serve as a rallying point for those interested in overcoming the aforementioned challenges and demonstrating the many benefits that can come from integrating physiological knowledge and tools into conservation science and resource management.

Looking to the future, there are a number of topical areas for which I would like to see more contributions. For example, invertebrates and plants remain poorly represented within the journal. In addition, there is an unfortunate lack of papers that discuss the success and failures of conservation physiology in terms of influencing policy or management. Of particular interest would be perspective articles that serve as case studies on different issues and what we could appropriately label as ‘Conservation Physiology in Action’, ideally written by policy makers and practitioners. Ecological models parameterized with physiological information (e.g. energy allocation, environmental thresholds) and used to predict responses of organisms to environmental change or to evaluate management scenarios are strongly encouraged. More studies that address some of the aforementioned challenges in terms of linking physiology to population-level processes through modelling (e.g. Fefferman and Romero, 2013) or experimental studies to establish cause and effect between stress and fitness (e.g. Thierry *et al.*, 2013) would be particularly valuable. I also see the notion of exploiting the physiology of invasive species to inform control methods as being an area ripe for conservation physiology to provide many breakthroughs. The development of the interface of disease ecology, physiology, and conservation is also timely. I would also like to be able to highlight more multi-disciplinary approaches that combine physiology with genetics, behaviour, and veterinary science, among the many other aspects of conservation science, recognizing the inherent need for integration (Balmford and Cowling, 2006). The inaugural article (i.e. Cooke *et al.*, 2013) provided a lengthy list of possible topics and that list still stands and essentially serves as the scope for the journal; please do check it out!

Ensuring broad knowledge mobilization and technology transfer between the producers of the knowledge and the receptor community (in this case, conservation practitioners) has always presented challenges to the conservation science community (Cook *et al.*, 2013). The journal *Conservation Physiology* has adopted a strategic position such that it is online only and fully open access. For the SEB as well as many researchers active in conservation physiology research, the notion of open access was a rather new one. Indeed, the other SEB journals are subscription based, as are most journals in conservation science, experimental biology, and physiology. However, the open access model is fitting, because it means that all of the material published will be easily and freely accessible by conservation practitioners, stakeholders, and the general public around the world, including those in developing countries. A recent analysis revealed that in conservation science, there was no evidence to support that open access articles were cited more than those articles behind a pay wall (Calver and Bradley, 2010); however, in applied realms, such as conservation, more important than citations is the ability of science to reach practitioners and influence policy and management. To establish a base of high-quality content to attract contributors, we are waiving publication fees entirely for the first 2 years (2013 and 2014) and inviting papers on pressing and topical issues from luminaries in conservation physiology. I hope you agree with me that the contents of the first volume represent diverse, high-quality content.

I am certainly thrilled with the state of the journal *Conservation Physiology* as we close the first volume and launch the second. Potential contributors (of individual articles or even special issues/theme sections) are welcome to contact me or other members of our talented and diverse editorial team. I must thank the authors, referees, editorial board, the plant editor (Dr Lawren Sack from UCLA), OUP, and SEB for unwavering support. The referees deserve particular recognition given that our average time to first editorial decision (for those papers that are sent to peer review) is <25 days. Indeed, in one instance I had three high-quality reviews in hand within 72 h of a paper being submitted. For me, the willingness of referees to provide thoughtful, constructive, and rapid reviews for a new journal is a testament to the excitement for the topic. *Conservation Physiology*—both the journal and the discipline—have an exciting future as we work collectively to generate the evidence base needed to understand and solve complex problems in conservation and resource management. I invite you to join us on this mission and welcome you to help to shape the journal and the discipline through contributing some of your best work to the journal *Conservation Physiology*.
